# The Hypothesis of a “Living Pulse” in Cells

**DOI:** 10.3390/life13071506

**Published:** 2023-07-04

**Authors:** Marina Walther-Antonio, Dirk Schulze-Makuch

**Affiliations:** 1 Department of Surgery, Division of Surgical Research, Mayo Clinic, Rochester, MN 55905, USA; waltherantonio.marina@mayo.edu; 2 Department of Obstetrics and Gynecology, Mayo Clinic, Rochester, MN 55905, USA; 3 Microbiome Program, Center for Individualized Medicine, Mayo Clinic, Rochester, MN 55905, USA; 4 Astrobiology Group, Center of Astronomy and Astrophysics, Technical University, 10623 Berlin, Germany; 5 German Research Centre for Geosciences (GFZ), Section Geomicrobiology, 14473 Potsdam, Germany; 6 Department of Plankton and Microbial Ecology, Leibniz Institute of Freshwater Ecology and Inland Fisheries, 16775 Stechlin, Germany; 7 School of the Environment, Washington State University, Pullman, WA 99164, USA

**Keywords:** motility, biosignature, intracellular movements

## Abstract

Motility is a great biosignature and its pattern is characteristic for specific microbes. However, motion does also occur within the cell by the myriads of ongoing processes within the cell and the exchange of gases and nutrients with the outside environment. Here, we propose that the sum of these processes in a microbial cell is equivalent to a pulse in complex organisms and suggest a first approach to measure the “living pulse” in microorganisms. We emphasize that if a “living pulse” can be shown to exist, it would have far-reaching applications, such as for finding life in extreme environments on Earth and in extraterrestrial locations, as well as making sure that life is not present where it should not be, such as during medical procedures and in the food processing industry.

## 1. Introduction

Life and motion are intrinsically related. All life forms move. Even if they do not have specific appendages for movement (which most life forms, even microbes, do) and are considered “non-motile”, they move due to their dynamic life processes that each living system must perform. Life requires compartmentalization to separate its insides from its external environment and life tries to achieve homeostasis by exchanging nutrients and wastes from the inside and the outside of the cell. These requirements will result in detectable changes and can be perceived as currents or as motions due to momentum conservation, changes in geometry, or changes in volume that occur within a single cell. This is the case even if life forms only adjust to their microenvironment around them and exchange solutes and gases to maintain intracellular equilibrium and disequilibrium to their outside environment. The exact amount of these changes is unknown at this time, and most changes are expected to lay below the detection limit. Investigating those would provide us with much desired insights into the internal working of a microorganism (and also on the possibility of the presence of a “living pulse”). We consider here the motion within life forms as a physical property and universal biosignature [1], which has the advantage of not being dependent on the given biochemistry of an organism and as such it also applies more broadly to life as we may not know it. 

The way an organism is moving in respect to its outside environment is termed motility and there are broad types of movements exhibited by microorganisms. Most familiar as a means of fast microbial movement (swimming and swarming) are flagella. Other microorganisms have pili that allow twitching and others glide through focal adhesions. Some are even non-motile or just move passively (Table 1). There is a huge diversity of microbial motility. In a recent review, it was claimed that there is a total of 18 different types of motility and that additional ones are expected to be discovered in the near future [2]. In fact, for most microbes, we do not even know how they move. Nevertheless, paths taken by microbes can be tracked with machine learning methods and the type of organism can be identified, in some cases down to the species level [3]. We do know, however, that motility is an early trait of the evolution of life that is present in all kingdoms of life [4,5,6]. Motility has recently been recognized as an important biomarker in astrobiological investigations [7,8] and specialized instrumentation such as holographic microscopy has been devised to detect it in a variety of environments [9,10]. While motility is the movement of a cell with respect to its outer environment, the intracellular motions will only be visible by high-resolution microscopy or more macroscopically when growth and reproduction occur.

## 2. The Hypothesis

There is motion within a cell from the myriad of internal processes and also at the cell boundaries when an organism interacts with its natural environment. Venturelli et al. [11] suggested that living organisms exhibit motion at the nanoscale that is above and beyond the frequency of Brownian motion such that it can be considered a universal signal of cellular life. Here, we designate the sum of these internal motions as a “living pulse” in analogy to the rhythmic pattern exhibited by complex organisms during breathing. Whether the “living pulse” is only a stochastic pattern resulting from the motions and adjustments to the above-mentioned changes or whether there is an intrinsic periodic pattern—perhaps as an emergent property of life compared to just chemical systems and in analogy to the pulse in more complex animals—is uncertain, but the below suggested investigations are hoped to reveal just that. We hypothesize that each microorganism has such a “living pulse”, a rhythmic pattern that in principle can be detected by state-of-the-art technology. 

Experimental evidence that such a “living pulse” exists comes from nanomechanical oscillators [12], which detect forces in the order of a piconewton and which were used to characterize living specimens and their metabolic cycles [13,14,15,16]. For example, cantilevers were used to investigate the activity of a cell’s molecular motors [17] and the particular vibrations of living *Saccharomyces cerevisiae* [18,19]. Cellular nanomotion has also been detected and monitored by micro- and nano-fabricated sensors [20,21,22,23] independent of cellular motility [11]. Extremely sensitive changes in mass and the metabolically induced oscillations of microorganisms have been measured using quartz crystal microbalances [24,25,26] and atomic force microscopy (AFM) [27,28], including metabolically induced oscillations of microorganisms [29,30], which support the notion that microbial metabolic activity could be utilized for life detection at the cellular scale [31,32]. The measured force by nanomechanical oscillators in the order of a piconewton [12] fares well with our estimate of the same order for the force required for one ion to go through a cellular membrane (about 2 piconewtons). This value is obtained by assuming a resting potential of 70 mV and an assumed thickness of 5 nm for a membrane. The equilibrium potential is then calculated using the Nernst equation, which is multiplied by a unit charge (1.6 × 10^−19^ C) to determine the amount of force needed. If we assume an ionic flux of 100 ions/sec through the membrane [33], we should be able to pick up a signal that required the force of at least 100 piconewtons/second. 

## 3. The Question of Detection

**Figure 1:**
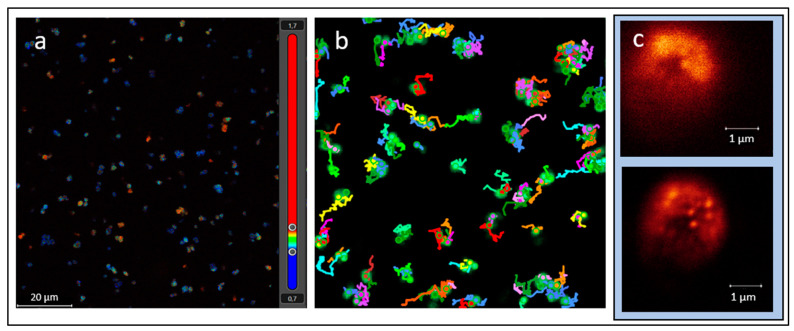
Images from a stimulated emission depletion (STED) microscope: (**a**) autofluorescence in *P. halocryophilus* with time-resolved contrast and color-coded average arrival times of photons (excitation frequency 495 nm, emission frequency 514–609 nm), (**b**) tracking of microbial motility in *P. halocryophilus* using convolutional microscopy, (**c**) convolutional microscopic image of *D. hansenii* dyed with Syto9 and with higher STED resolution (below) improving the visibility of organelles and internal structure.

While we interpret the hypothetical “living pulse” of a microorganism to be the sum of the internal processes occurring and the interactions of the membrane with the outer environment, especially movements across the ion channels, the magnitude is still expected to be miniscule. However, new microscopes, such as stimulated emission depletion (STED) microscopes, allow the observation of the movement of organelles or vesicles within the cell and also pick up autofluorescence in the cell and thus will at least allow us to arrive at better estimates at which frequency and magnitude a signal pattern could be expected (Figure 1). An overview of a sample cell can be obtained with a convolutional microscope. AI software is then employed to identify rare and anomalous observations, which are then further scrutinized with STED microscopy at a super-resolution of 50 nm or below [34]. The maximum frame rate per second is about 30 for the imaging of living organisms, which might be in the detection range to detect the “living pulse”. This would allow us to visualize changes in cell structure and fluorescent markers associated with cellular processes such as signaling or contractions. If a fluorescent marker is used to label a specific protein or organelle within the cell that is hypothesized to change in response to the “living pulse”, STED microscopy could be used to visualize these changes with high spatial resolution to detect the presence of a pattern. In addition, it will be useful to monitor any morphological changes at these magnifications. For example, if cells are used without a rather rigid cell wall, vibrational patterns might be identified at the cell membrane. 

A complementary technique to use would be scanning ion-conductance microscopy (SICM), which is a non-invasive scanning technique employed to study dynamic cellular processes at the nanoscale, particularly those that are related to ion conductance [35]. One specific approach we propose is to use a dead cell as a control, observe it for a specific time period, and record any instrument “flickers”. Then, use a living cell, observe and record its life field view from which the white noise of the dead cell is subtracted. Software could be used to remove particular wavelength periodicities to reveal any intrinsic pattern to the cell: the “living pulse”.

The “living pulse” hypothesized here is not to be mistaken with the circadian rhythm, which was not only found in eukaryotes [36] but has also been detected in cyanobacteria [37,38,39]. It is thought to be exhibited in cyanobacteria due to a selective advantage of cyanobacteria being adapted to the light–dark cycle [40]. Thus, a circadian rhythm is an organism’s response to environmental cycles in contrast to the “living pulse”, which is thought to be an inherent rhythmic pattern to a microbial organism. A circadian rhythm may also exist in the purple non-sulfur bacterium *Rhodopseudomonas palustris* and in *Bacillus subtilis* based on gene expression patterns [41,42] and has also been proposed for the human microbiome [43], but it is unclear which or whether all microbes have a circadian rhythm. While the circadian rhythm is unrelated to the “living pulse” being an adaptation to environmental cues, the “living pulse” may be more pronounced during times of higher activity, such as during the light cycle in cyanobacteria. 

Even if the detection range is not achieved by the above methodology alone, there are additional options to enhance the potential signal. First, microfluidic platforms could be used to separate single cells and coat them with a hydrogel matrix or to fix them with optical laser tweezers. Cells used for initial trials would not have a cell wall, which might dampen the signal. Moreover, if the ion channels are determined to be a significant contributor to the overall signal, then genetic modifications of the tested species through evolutionary generation experiments might be warranted to maximize the number of ion channels in a specific tested species. Another, probably easier, approach is to enhance or amplify the signal by using stimulants such as L-serine [44,45]. Alternatively, other stimulants such as heat, oxygen, or light could be used to increase the signal strength.

One challenge will be to distinguish the hypothetical “living pulse” from environmental noise. There are many processes that could lead to environmental noise. They include for example chemical concentration gradients, physical disturbances, and even the interaction of one organism with another one. However, all environmental noises have the commonality that they originate from outside the cells. Thus, the direction of the rhythmic pattern can be used as a distinguishing marker. If the pattern is detected moving from the inside of the cell to the outside, we interpret it to be the “living pulse”, because environmental noise would travel from the outside of the cell to the inside. If there is a periodic pattern, it can be detected in a controlled environment where abiotic periodicities are either absent or known. While we hypothesize that all living microbes will exhibit a “living pulse”, we expect the frequency and the magnitude to be different depending on the species just as is the case for animals.

## 4. The Significance of Detecting a “Living Pulse”

If the hypothesized “living pulse” can be detected, it would have far-reaching applications. While most of Earth´s surface areas are populated by microbes, there are extreme environments where this is questionable. This includes areas in the hyperarid Atacama Desert [46,47], the Don Juan Pond in Antarctica [48,49], the Dallol Geothermal Area in Ethiopia [50,51], and newly created volcanic landscapes [52]. The “living pulse” would also be an ideal tool to determine whether life exists on an extraterrestrial body. The Viking life detection experiments conducted on Mars, the only life detection experiments ever conducted on an extraterrestrial body, are underlining this problem as it still has not been resolved whether life was actually detected or not [53,54,55]. Moreover, given concurrent missions to Mars and especially given the expected sample return missions from Mars to Earth by both NASA [56] and China [57] in the early 2030s, a universal biosignature independently of a life form´s specific biochemistry is urgently needed to satisfy planetary protection concerns. This is particular important for backward contamination in order to safeguard Earth´s biosphere. Furthermore, there are locations and places where we do not want life to be present and the “living pulse” could be used to verify that. Examples are on surgery tables during medical procedures, including the instruments utilized, and during food processing. The detection of *Deinococcus radiodurans*, which was discovered because it survived the application of high doses of gamma radiation to sterilize canned food [58], shows that sterile conditions cannot be guaranteed even if sterilizing stressors are applied. 

## 5. Conclusions

While the existence of a “living pulse” in microorganisms remains unexplored, if such a signal can be detected, it will have profound consequences as a universal biosignature independent of a microorganism´s biochemistry. It would be an invaluable tool for us to find life in extreme environments on Earth and in extraterrestrial environments beyond Earth, including when enforcing planetary protection protocols. The detection of this physical property of life could also have important implications on Earth, such as in the detection of viable microorganisms in the medical field and in food processing.

## Figures and Tables

**Table 1:** Types of microbial motility.

Type of Movement	Description	Example Organisms
Swimming	Movement of an individual organism, powered by rotating flagella or archaella	Many bacteria and archaea such as *Escherichia coli*, *Bacillus subtilis*, *Vibrio cholerae*, *Halobacterium salinarum*, *Methanococcus voltae*
Twitching	A form of crawling to move over a surface using a type IV pilus to pull a cell forward, similar to throwing a hook and pulling the organisms in that direction	*Acinetobacter calcoaceticus*, *Pseudomonas aeruginosa*, *Shewanella putrefaciens*, *Vibirio cholerae*
Gliding	Movement along the surface of aqueous films without the aid of external appendages such as flagella, cilia, or pili	Certain rod-shaped bacteria, e.g., myxobacteria such as *Myxococcus xanthus*
Sliding	Passive movement along for example a concentration gradient or by the presence of surfactants	*B. subtilis*, *Serratia marcescens*, *P. aeruginosa*
Non-motile	Growing only along a stab line when cultured	Pathogenic bacteria, such as *Streptococcus* sp., *Klebsiella pneumoniae*, and *Yersinia pestis*, but also for example *Deinococcus radiodurans*
Reproduction	Cell duplication	All life forms
Swarming	Rapid (2–10 μm/s) and coordinated translocation of a bacterial population across a solid or semi-solid surface	*Proteus mirabilis*, *E. coli*, *B. subtilis*, *P. aeruginosa*

Note: Some microbes (e.g., *B. subtilis*) have been characterized as exhibiting different modes of motility.

## Data Availability

No additional data available.
